# Atorvastatin impairs liver mitochondrial function in obese Göttingen Minipigs but heart and skeletal muscle are not affected

**DOI:** 10.1038/s41598-021-81846-9

**Published:** 2021-01-26

**Authors:** Liselotte Bruun Christiansen, Tine Lovsø Dohlmann, Trine Pagh Ludvigsen, Ewa Parfieniuk, Michal Ciborowski, Lukasz Szczerbinski, Adam Kretowski, Claus Desler, Luca Tiano, Patrick Orlando, Torben Martinussen, Lisbeth Høier Olsen, Steen Larsen

**Affiliations:** 1 The LIFEPHARM Centre, Department of Veterinary and Animal Sciences, Faculty of Health and Medical Sciences, University of Copenhagen, Ridebanevej 9, 1870 Frederiksberg, Denmark; 2 Xlab, Center for Healthy Aging, Department of Biomedical Sciences, Faculty of Health and Medical Sciences, University of Copenhagen, Blegdamsvej 3B, 2200 Copenhagen, Denmark; 3 Global Drug Development, Novo Nordisk A/S, Novo Nordisk Park, 2760 Måløv, Denmark; 4 Clinical Research Centre, Medical University of Bialystok, 15-089 Białystok, Poland; 5 Center for Healthy Aging, Department of Cellular and Molecular Medicine, University of Copenhagen, Blegdamsvej 3B, 2200 Copenhagen, Denmark; 6 Department of Life and Environmental Sciences (DISVA), Polytechnic University of Marche, via Brecce Bianche, Ancona, Italy; 7 Department of Public Health, Faculty of Health and Medical Sciences, University of Copenhagen, Øster Farimagsgade 5, 1014 Copenhagen, Denmark

**Keywords:** Cardiovascular biology, Experimental models of disease, Dyslipidaemias, Cell biology, Mitochondria, Energy metabolism

## Abstract

Statins lower the risk of cardiovascular events but have been associated with mitochondrial functional changes in a tissue-dependent manner. We investigated tissue-specific modifications of mitochondrial function in liver, heart and skeletal muscle mediated by chronic statin therapy in a Göttingen Minipig model. We hypothesized that statins enhance the mitochondrial function in heart but impair skeletal muscle and liver mitochondria. Mitochondrial respiratory capacities, citrate synthase activity, coenzyme Q10 concentrations and protein carbonyl content (PCC) were analyzed in samples of liver, heart and skeletal muscle from three groups of Göttingen Minipigs: a lean control group (CON, n = 6), an obese group (HFD, n = 7) and an obese group treated with atorvastatin for 28 weeks (HFD + ATO, n = 7). Atorvastatin concentrations were analyzed in each of the three tissues and in plasma from the Göttingen Minipigs. In treated minipigs, atorvastatin was detected in the liver and in plasma. A significant reduction in complex I + II-supported mitochondrial respiratory capacity was seen in liver of HFD + ATO compared to HFD (*P* = 0.022). Opposite directed but insignificant modifications of mitochondrial respiratory capacity were seen in heart versus skeletal muscle in HFD + ATO compared to the HFD group. In heart muscle, the HFD + ATO had significantly higher PCC compared to the HFD group (*P* = 0.0323). In the HFD group relative to CON, liver mitochondrial respiration decreased whereas in skeletal muscle, respiration increased but these changes were insignificant when normalizing for mitochondrial content. Oral atorvastatin treatment in Göttingen Minipigs is associated with a reduced mitochondrial respiratory capacity in the liver that may be linked to increased content of atorvastatin in this organ.

## Introduction

Ischemic heart disease and stroke are the leading causes of death globally, according to the World Health Organization^1^. Hypercholesterolemia is known to be a major risk factor for developing cardiovascular disease^2^. Statins (3-hydroxy-3-methyl-glutaryl-coenzyme A inhibitors) are the cornerstone in the management of dyslipidemia^3^. In the US and in Denmark, approximately 20% of people above 40 years of age have been treated with statins^4,5^. Besides their cholesterol lowering effects, statins are known to exert other clinical actions that can be either beneficial or harmful in the cardiovascular and other organ systems^6,7^. Although statins are usually well tolerated, the most common adverse reaction is myalgia^8^.

In cardiac muscle, atorvastatin has been shown to improve systolic function and reduce inflammation in human patients with non-ischemic heart failure^9^. Simvastatin was shown to preserve diastolic function of the heart by reducing cardiac muscle remodeling in a minipig model of the metabolic syndrome^10^.

According to cross-sectional studies, statins are relatively rare causes of drug-induced liver injury in the general population but the outcome can be fatal^11^. Clinicians seem reluctant to prescribe statins in patients with non-alcoholic fatty liver disease (NAFLD)^12–14^ despite increasing evidence showing that statins possess anti-inflammatory, anti-fibrotic and anti-oxidative stress properties in NAFLD^15^. Their use for this indication is still debated, possibly due to the yet unrecognized underlying mechanisms^16^.

The subcellular mechanisms behind the non-cholesterol lowering effects exerted by statins in various organ systems are currently not known. It also remains unknown to what extent statins concentrate in different tissue types after chronic therapy. However, in vivo studies, indicate that the use of statins is associated with impairment of mitochondrial function [mitochondrial respiratory capacity and reactive oxygen species (ROS) production] in skeletal muscle^17–20^. Statin treatment has also been shown to reduce skeletal muscle mitochondrial content and concentrations of the mitochondrial coenzyme ubiquinone (Q10)^17,18^.

It has been proposed that statins modify the mitochondrial function in skeletal muscle and heart muscle in an opposite directed manner^21,22^. In intact skeletal muscle cells, acute treatment with simvastatin impaired the mitochondrial respiratory capacity while in cardiomyocytes the mitochondrial respiration was preserved^21^. Bouitbir et al. demonstrated in vitro that atorvastatin treatment elicited an enhanced respiratory capacity of mitochondria with a low but significant (+ 53%) increase in mitochondrial H_2_O_2_ emission in rat cardiomyocytes. In skeletal muscle cells, a multitude greater (368%) increase in H_2_O_2_ emission was observed alongside with impaired mitochondrial respiration^22^. The authors proposed the tissue dependent opposite directed effects on the mitochondria as a unifying mechanism responsible for adverse and beneficial clinical effects seen in skeletal muscle and the cardiovascular system, respectively after use of statins. To the best of our knowledge, the opposite directed effect of statins in heart and skeletal muscle has not yet been shown in vivo at doses corresponding to the therapeutically relevant dose of atorvastatin of 10–80 mg per day in adults for the reduction of cardiovascular risk^23^.

The aim of this study was to investigate how treatment with atorvastatin in a therapeutically relevant dose influences the mitochondrial function in the three energy-demanding tissues: liver, heart and skeletal muscle from the same animal, using a Göttingen Minipig model. We hypothesized that the mitochondrial respiratory capacity is enhanced in heart muscle and impaired in skeletal muscle. In line with the prior results^22^, we hypothesized that atorvastatin treatment enhances H_2_O_2_ release and increases the level of oxidative stress in cardiac and skeletal muscle but with different orders of magnitude, with skeletal muscle generating a greater H_2_O_2_ release than cardiac muscle. Finally, we investigated the hypothesis that atorvastatin depletes the concentration of the mitochondrial cofactor and antioxidant Q10 in tissues.

We took advantage of a cohort of Göttingen Minipigs being available in conjunction with a larger study. Selected data, including basic characteristics of the Göttingen Minipigs used in this study, have therefore been published previously elsewhere for other scientific purposes^24–28^. One group of minipigs was induced with obesity through a diet-intervention and a subset of the obese minipigs was superimposed with chronic atorvastatin treatment. The diet-induced obese Göttingen Minipig is well characterized as a model for the human metabolic syndrome displaying severe obesity with dyslipidemia, coronary and aortic atherosclerosis and NAFLD^25,29^.

To discern the proportion of changes in mitochondrial function attributed to obesity from changes exerted by statin treatment in each of the three tissue types, a lean control group was included as a non-obese reference group. A secondary aim of the study was therefore to test the hypothesis that the presence of obesity and dyslipidemia in minipigs mediates tissue specific changes of mitochondrial content and function in the highly energy demanding tissues: liver, heart and skeletal muscle.

## Results

### Animal characteristics

The Göttingen Minipig represents a large animal model featuring several similarities to humans in cardiovascular anatomy, muscle physiology and the ability to develop metabolic syndrome after eating a Western-like diet^29^.

At study start, the body weight (BW) was comparable between the lean control minipigs (CON, n = 6), the minipigs being fed a high fat diet (HFD, n = 7) and the minipigs being fed high fat diet superimposed with atorvastatin treatment (HFD + ATO, n = 7). Table 1 provides an overview of body composition, gross weight of organs and concentrations of circulating triglycerides, total cholesterol, alanine transaminase, alkaline phosphatase, aspartate transaminase and creatine kinase in each group of minipigs. The HFD + ATO group did not differ from the HFD group in any of the parameters. During the study period the HFD and HFD + ATO animals increased their body weight progressively compared to CON animals (Supplementary material Fig. S1). At termination, the HFD (*P* = 0.003) and HFD + ATO (*P* = 0.003) groups had significantly higher body weight (BW) than CON and the body fat % was significantly increased in HFD (*P* < 0.0001) and HFD + ATO (*P* < 0.0001) groups compared to CON.

**Table 1:** Basic characteristics in the study population of Göttingen Minipigs.

Minipig characteristics	CON	HFD	HFD + ATO	Overall *P* value
*n*	*6*	*7*	*7*	
Body weight at study start (kg)	16.5 (15.5–17.5)	17.0 (16.0–18.0)	17.0 (16.0–19.0)	0.83
Body weight at termination (kg)	39.5 (37.9–41.1)	77.0 (69.0–81.0)^a^	69.5 (65–76.5)^a^	0.0016^#^
Heart weight (g)	134.5 (120.3–143.3)	190.0 (181.0–223.0)^a^	196.0 (193.0–221.0)^a^	< 0.0001^##^
Body fat (%)	27.4 (23.9–32.1)	63.6 (59.0–66.6)^a^	64.4 (57.0–67.5)^a^	< 0.0001
Liver weight (g)	513.5 (466.3–585.0)	1908.0 (1067.0–2150.0)^a^	2045.0 (1960.0–2373.0)^a^	0.0018^#^
Triglyceride (mmol/L)	0.34 ^(n=5)^ (0.27–0.35)	0.61 (0.52–1.49)^a^	0.77 ^(n=6)^ (0.35–0.80)^a^	0.025^#^
Total cholesterol (mmol/L)	1.70 (1.34–2.18)	11.94 (9.09–17.99)^a^	9.43 (4.92–11.11)^a^	0.0021^#^
ALP (U/L)	158.0 (109.5–216.0)	450.0 (335.0–661.0)^a^	664.0 (515.0–777.0)^a^	< 0.0001^##^
ALAT (U/L)	43.0 (39.3–50.0)	30.0 (24.0–33.0)^a^	29.0 (25.0–30.0)^a^	0.0007
ASAT (U/L)	32.5 (21.0–56.0)	32.0 (22.0–36.0)	36.0 (31.0–39.0)	0.99^##^
CK (U/L)	416.5 (211.0–808.5)	180.0 (122.0–272.0)^a^	161.0 (115.0–205.0)^a^	0.012^##^

*ALP* alkaline phosphatase, *ALT* alanine-transaminase, *ASAT* aspartate transaminase, *CK* creatine kinase. Bodyfat (%) and triglyceride were determined 1–2 weeks prior to termination. Other parameters were determined at termination of the minipigs when not stated otherwise.

Data are presented as median (interquartile range). ^a^Different from CON, ^#^Non-parametric test, ^##^Log transformation of data was performed before statistical analyses.

There were no statistically significant differences between the HFD and HFD + ATO groups in background characteristics.

At termination, minipigs in the HFD and HFD + ATO groups had significantly higher weight of the heart (*P* < 0.0001 and *P* < 0.0001, respectively) and the liver (*P* = 0.0034 and *P* = 0.0034, respectively) compared to CON. The total circulating concentration of cholesterol was significantly increased in HFD (*P* = 0.0034) and HFD + ATO (*P* = 0.0034) groups compared to CON but atorvastatin treatment did not significantly change total cholesterol concentrations (*P* = 0.44 HFD + ATO vs. HFD, Table 1).

### Atorvastatin content in tissue and circulation

**Figure 1:**
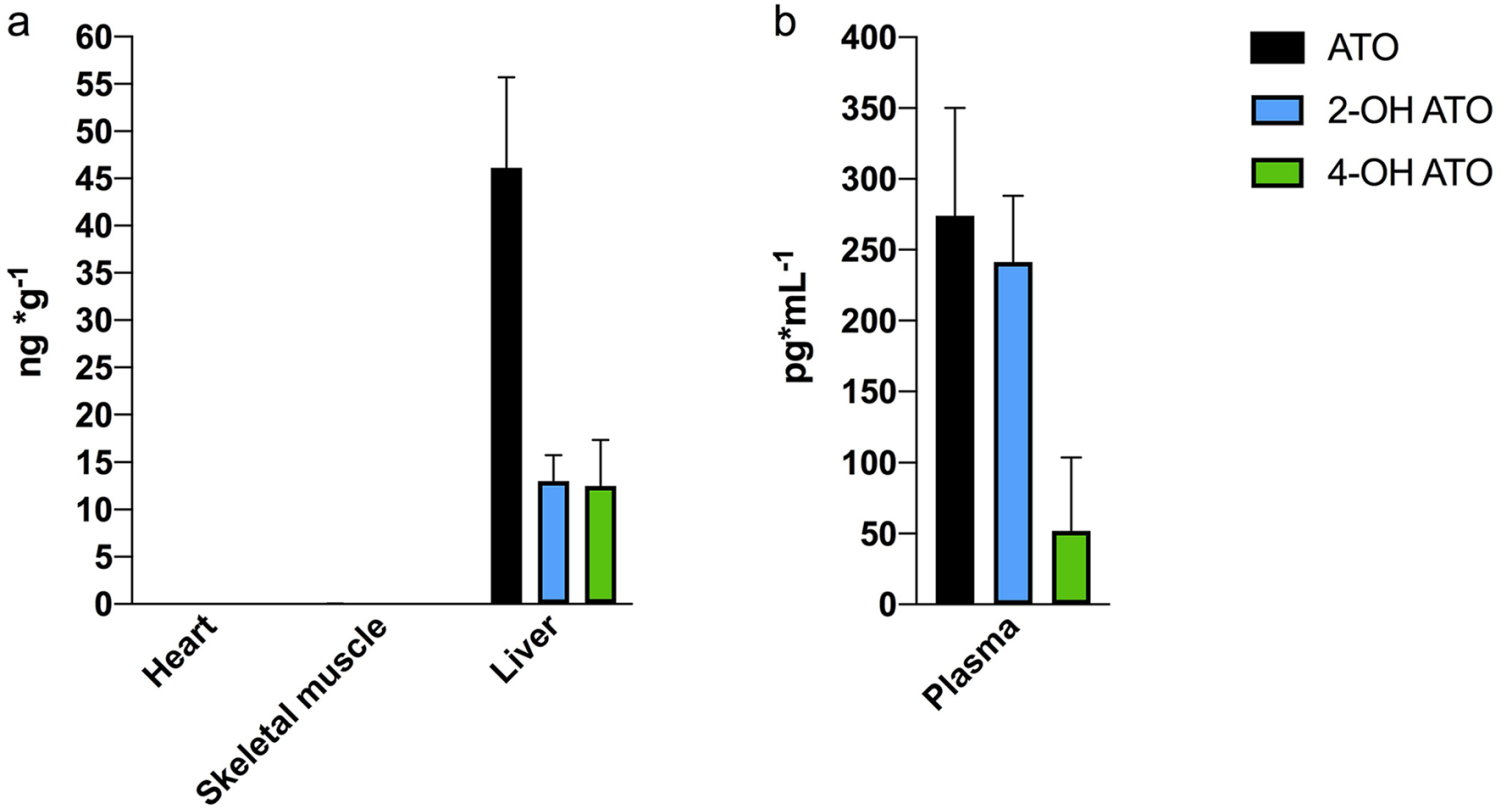
Concentrations of atorvastatin and active metabolites of atorvastatin in Göttingen minipigs treated with atorvastatin (HFD + ATO, n = 7). (**a)** Tissue concentrations (ng/g) analysed in heart muscle, skeletal muscle and liver, of atorvastatin calcium salt (ATO) and two metabolites; 2-hydroxy atorvastatin dihydrate monosodium salt (2-OH ATO) and 4-hydroxy atorvastatin disodium salt (4-OH ATO). The liver was the only tissue with detectable concentrations. (**b)** Plasma concentrations (pg/mL) of ATO, 2-OH ATO and 4-OH ATO) in the minipigs. Data are means ± SEM.

We used ultrahigh-performance liquid chromatography-mass spectrometry (UHPLC-MS) to measure atorvastatin calcium salt and its two active metabolites: 2-hydroxy atorvastatin dihydrate monosodium salt (2-OH ATO) and 4-hydroxy atorvastatin disodium salt (4-OH ATO) in tissue samples from liver, heart, skeletal muscle and in plasma of all the minipigs. In plasma and liver from atorvastatin treated minipigs but not in heart and skeletal muscle, atorvastatin calcium salt, 2-OH ATO and 4-OH ATO were detected (Fig. 1). In one minipig treated with atorvastatin, only 2-OH ATO and 4-OH ATO were detected in liver but not atorvastatin calcium salt. No quantifiable concentrations of atorvastatin and metabolites could be detected in tissue samples or in plasma in the CON and HFD groups.

### Mitochondrial content marker

**Figure 2:**
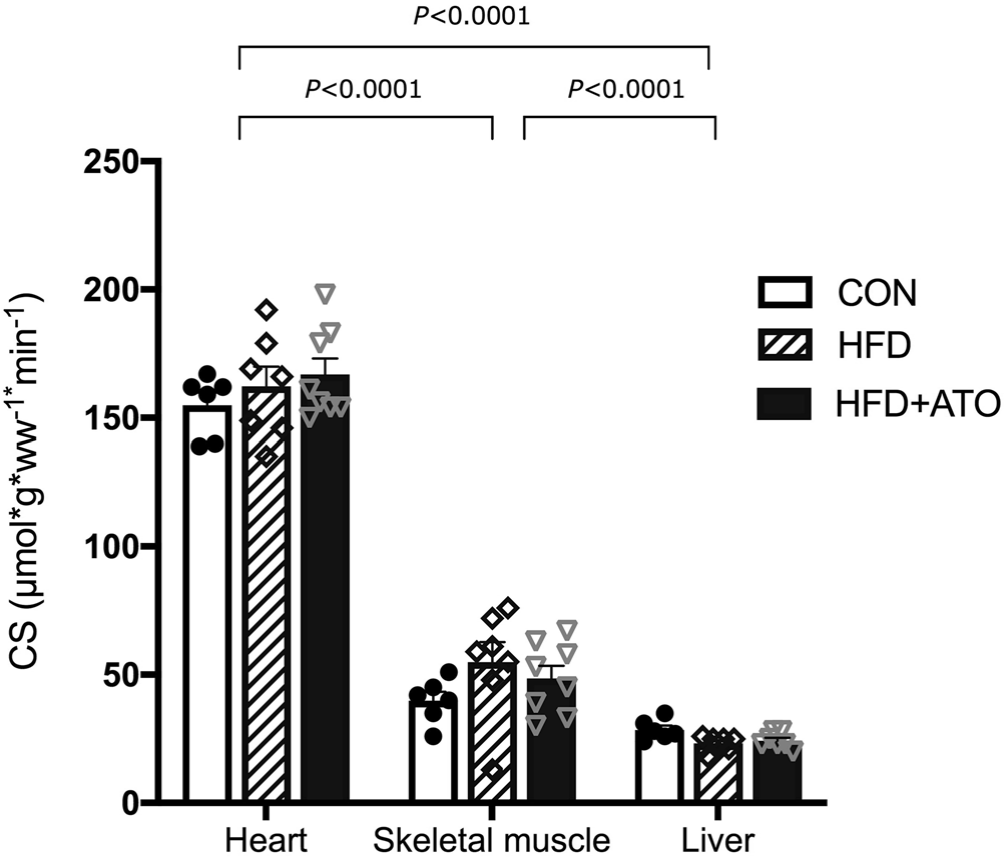
Citrate synthase (CS) activity (marker of mitochondrial content) measured by spectrophotometry. Mitochondrial content was determined in heart muscle, skeletal muscle and liver in control (CON), obese (HFD) and obese atorvastatin treated Göttingen Minipigs (HFD + ATO). CS activity was significantly higher in heart muscle compared to skeletal muscle and liver (*P* < 0.0001). Skeletal muscle had significantly higher CS activity than liver (*P* < 0.0001. Among the groups there were no significant differences in CS activity neither in heart muscle, skeletal muscle nor in the liver. Data are reported as single measurements, bars and error bars denote means ± SEM, n = 6–7 in each group.

We measured citrate synthase (CS) activity as a surrogate marker of mitochondrial content and used this marker to normalize all parameters of respiratory capacities to mitochondrial content. There was no difference in CS activity between the CON, HFD and HFD + ATO groups in any of the three tissue types, indicating that neither treatment with atorvastatin nor obesity itself modified the content of mitochondria in liver, heart and skeletal muscle (Fig. 2). Heart muscle had the highest content of CS activity compared to skeletal muscle (*P* < 0.0001) and liver (*P* < 0.0001). The skeletal muscle had significantly higher CS activity than liver (*P* < 0.0001, Fig. 2).

### Coenzyme Q10 concentrations

**Figure 3:**
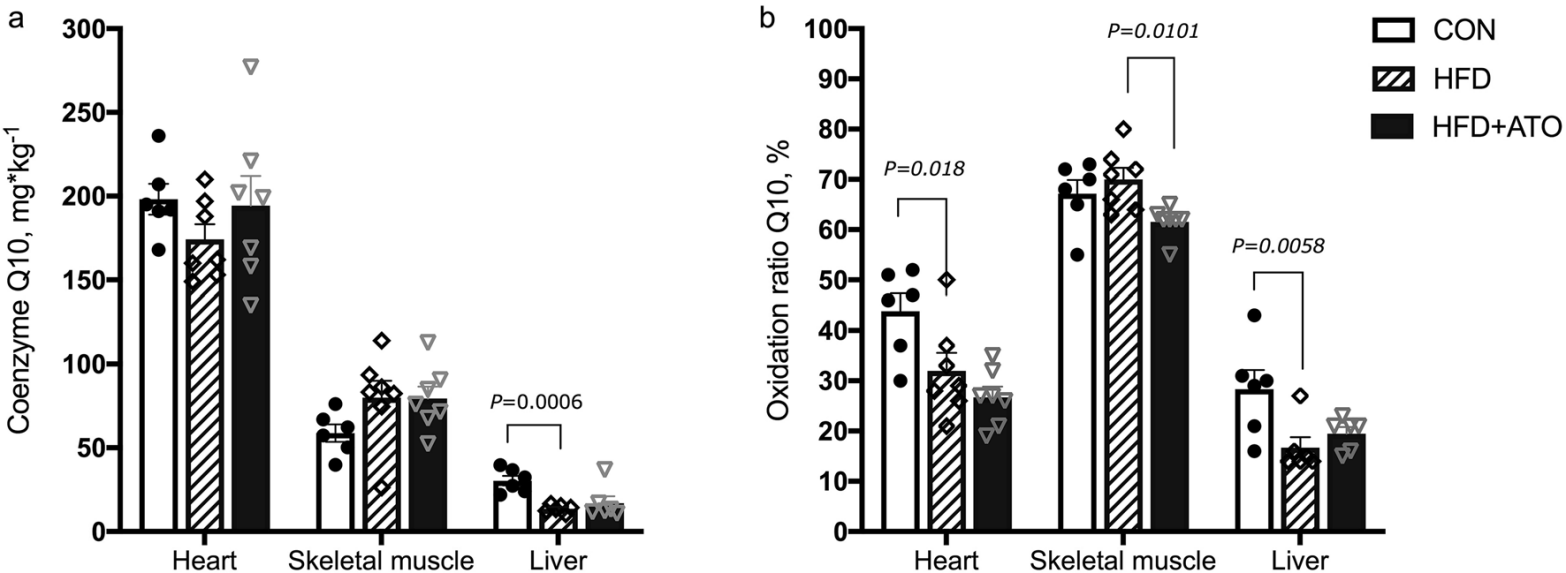
Total concentration and oxidation rate (%) of Coenzyme Q10 (Q10) in heart muscle, skeletal muscle and liver in control (CON), obese (HFD) and obese atorvastatin treated Göttingen Minipigs (HFD + ATO). Q10 oxidation rate was calculated as the percentage of total Q10 that was oxidized. (**a**) Total Q10 concentration in heart muscle, skeletal muscle and liver from the Göttingen Minipigs. (**b**) Oxidation rate of Q10 in heart muscle, skeletal muscle and liver from the Göttingen Minipigs. Bars and error bars denote means ± SEM, n = 6–7 in each group.

Concentration of coenzyme Q10 (Q10) and the proportion (%) of oxidized Q10 relative to total Q10 (oxidation rate) were determined in tissue samples from heart, skeletal muscle and liver. The heart was most abundant in total Q10 followed by skeletal muscle and liver (Fig. 3a).

There were no observed significant differences in the total Q10 concentration between the CON, HFD and HFD + ATO groups in heart and skeletal muscle. In the liver, the HFD group had significantly lower Q10 content compared to the CON group (*P* < 0.0006, Fig. 3a). The Q10 concentration in liver was comparable between the HFD and the HFD + ATO groups. Significant improvements in the oxidative status of Q10 were observed in HFD compared to CON group in heart muscle (*P* = 0.018), in HFD + ATO compared to HFD in skeletal muscle (*P* = 0.0101) and in HFD compared to CON in liver (*P* = 0.0058, Fig. 3b).

### Mitochondrial respiratory capacity

#### Liver

**Figure 4:**
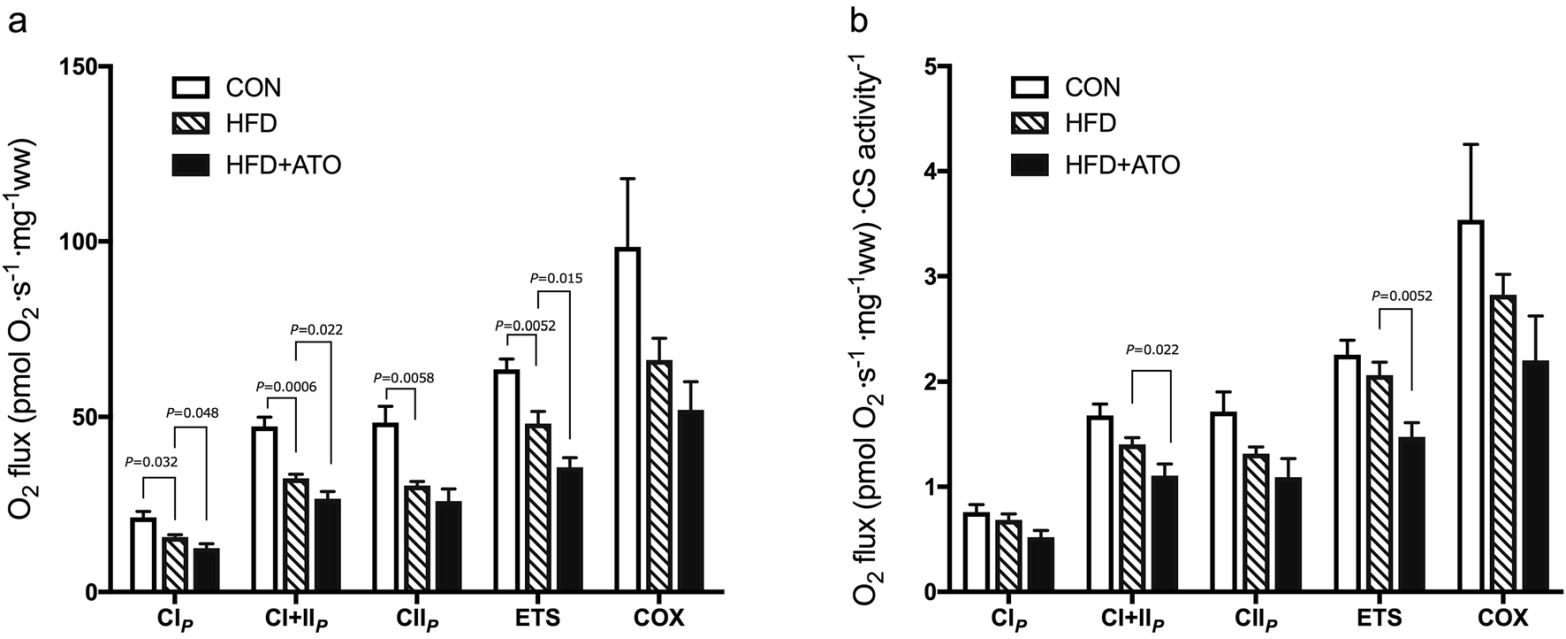
Effect of obesity and atorvastatin treatment on mitochondrial respiratory capacity in liver of Göttingen Minipigs. (**a)** Maximal mitochondrial respiratory capacity expressed as respiratory flux per mg tissue with substrates and inhibitors stimulating electron flow through complex I (CI_*P*_*:* malate + glutamate), complex I + II (CI + II_*P*_: + succinate*)*, complex II (CII* P* + rotenone), maximal uncoupled respiration (ETS + FCCP) and cytochrome c oxidase (COX: antimycin A + ascorbate + TMPD) activity in the liver of lean control minipigs (CON), obese minipigs (HFD) and obese minipigs treated with atorvastatin (HFD + ATO). The substrate and inhibitor titration protocols are described in detail in supplementary material. (**b**) Mitochondrial respiratory capacities shown in A in the liver normalized to CS activity. *ETS* electron transport system capacity, *FCCP* carbonyl cyanide *p*-(triflouromethoxy) phenylhydrazone, *TMPD* *N*,*N*,*N*′,*N*′-Tetramethyl-*p*-phenylediamine. Data are means ± SEM, n = 6–7 in each group.

An important finding of the study was a reduced mitochondrial respiratory capacity in the liver of minipigs treated with atorvastatin compared to obese minipigs (Fig. 4). State 3 respiration supported with complex I substrates was significantly reduced in the HFD + ATO group compared to the HFD group (CI_P_, *P* = 0.048, Fig. 4a). State 3 respiration with convergent electron flow through complex I + II was also significantly reduced in HFD + ATO compared to HFD group (CI + II_P_, *P* = 0.022, Fig. 4a) and following uncoupling the respiratory capacity remained significantly lower in HFD + ATO compared to HFD minipigs (ETS, *P* = 0.015, Fig. 4a). State 3 respiration supported solely with complex II substrate (CII_P_) as well as cytochrome c oxidase (COX) activity were numerically reduced in the HFD + ATO group compared to the HFD group but the differences did not reach statistical significance (Fig. 4a).

After normalization of the respiratory flux to CS activity, respiration supported with complex I + II substrates (CI + II_P_, *P* = 0.022) and uncoupled respiration (ETS, *P* = 0.0052) remained significantly reduced in HFD + ATO compared to HFD (Fig. 4b). Normalized state 3 respiration supported with complex I substrates was insignificantly reduced in HFD + ATO compared to HFD (CI_P_, Fig. 4b).

An increase in respiration rate in liver from state 3 supported with complex I + II substrates to respiration after uncoupling with the proton ionophore carbonyl cyanide *p*-(triflouromethoxy)phenylhydrazone (FCCP) was observed graphically (ETS, Fig. 4a). This finding indicates the presence of a reserve oxidative phosphorylation capacity of liver mitochondria in minipigs, restricted by the phosphorylation system.

Significant reductions of the respiratory capacity in liver mitochondria were observed in the HFD compared to CON minipigs in state 3 respiration supported with complex I substrate (*P* = 0.032), complex I + II substrate (*P* = 0.0006), complex II substrate (*P* = 0.0058) and in uncoupled respiration (ETS, *P* = 0.0052, Fig. 4a). When normalizing for CS activity, the respiratory capacities were numerically reduced in HFD compared to CON but statistical significance was not reached in any of the substrate states (Fig. 4b).

#### Skeletal muscle

**Figure 5:**
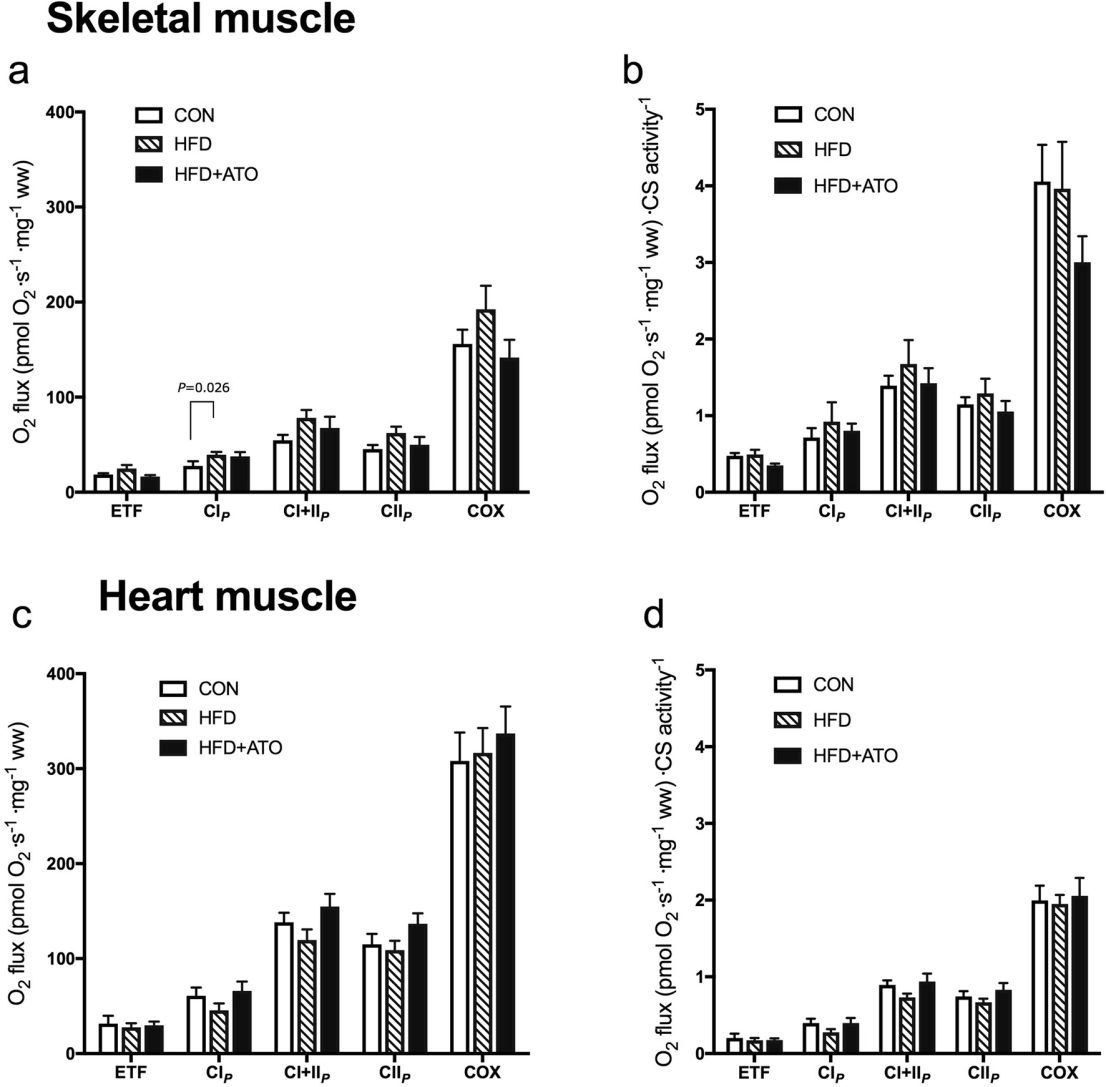
Effect of obesity and atorvastatin treatment on mitochondrial respiratory capacity in skeletal muscle and in heart muscle from Göttingen Minipigs. (**a**) Mitochondrial respiratory flux per mg tissue in permeabilized skeletal muscle fibers of lean control minipigs (CON), obese minipigs (HFD) and obese minipigs treated with atorvastatin (HFD + ATO). The respiratory states shown are: State 3 respiration supported by complex I substrates (CI_*P*_: malate + glutamate + ADP), complex II substrate (CII_*P*_: + succinate) followed by respiratory flux after inhibition of complex I (CII_*P*_: + rotenone), stimulation of cytochrome c oxidase activity (COX: antimycin A + ascorbate + TMPD. Moreover, state 3 respiration supported with complex I substrates and fatty acid (ETF: malate + glutamate + palmitoyl carnitine). The protocols are described in detail in supplementary material. (**b**) The mitochondrial respiratory capacities from A normalized to CS activity in skeletal muscle. (**c**) Mitochondrial respiratory flux per mg tissue in permeabilized heart muscle fibers using the same substrate states as shown in A in lean control minipigs (CON), obese minipigs (HFD) and obese minipigs treated with atorvastatin (HFD + ATO). (**d**) Mitochondrial respiratory capacities normalized to CS activity in heart muscle. *ETF* electron transfer flavoprotein, *TMPD* *N*,*N*,*N′*,*N′*-Tetramethyl-*p*-phenylediamine. Data are means ± SEM, n = 6–7 in each group.

In skeletal muscle, the mean respiratory capacity in all the measured substrate states was numerically lower in HFD + ATO minipigs compared to HFD but the differences did not reach statistical significance (Fig. 5a).

When comparing HFD with CON minipigs the mitochondrial respiration was increased in the HFD group in each substrate state (Fig. 5a) but statistical significance was reached only in state 3 respiration supported with complex I substrates (CI_P_, *P* = 0.026, Fig. 5a). State 3 respiration supported with complex I + II showed a tendency (CI + II_P_, *P* = 0.063) for increased respiratory capacity (Fig. 5a). When normalizing for CS activity the numerical increase in mitochondrial respiration supported with complex I and complex I + II substrates in HFD compared to CON minipigs remained but no significant differences were identified (Fig. 5b).There was no numerical increase in the mitochondrial respiratory capacity after uncoupling with FCCP, indicating that the phosphorylation system exerts no limitation on the capacity of mitochondrial oxidative phosphorylation in the skeletal muscle of the minipigs (data not shown).

#### Heart

In heart muscle there was a tendency towards an increased state 3 respiratory capacity supported with complex I + II substrates (CI + II_P_, *P* = 0.06) and complex II substrate (CII_P_, *P* = 0.09) in HFD + ATO compared to HFD (Fig. 5c). When normalizing for CS activity, the mitochondrial respiratory capacities in the heart remained numerically higher in HFD + ATO compared to HFD but the difference did not reach statistical significance in any of the substrate states (Fig. 5d).

When comparing HFD with the CON group, state 3 mitochondrial respiratory capacity supported with complex I, complex I + II, complex II and fatty acid substrate was numerically but not significantly reduced in HFD relative to CON minipigs (CI_P,_ CI + II_P_, CII_P,_ ETF, Fig. 5c). This remained after normalizing the respiratory capacity for CS activity (Fig. 5d). Similar to skeletal muscle, there was no effect of titration with the uncoupler FCCP on the maximal respiratory capacity in heart muscle (data not shown).

### H_2_O_2_ release

**Figure 6:**
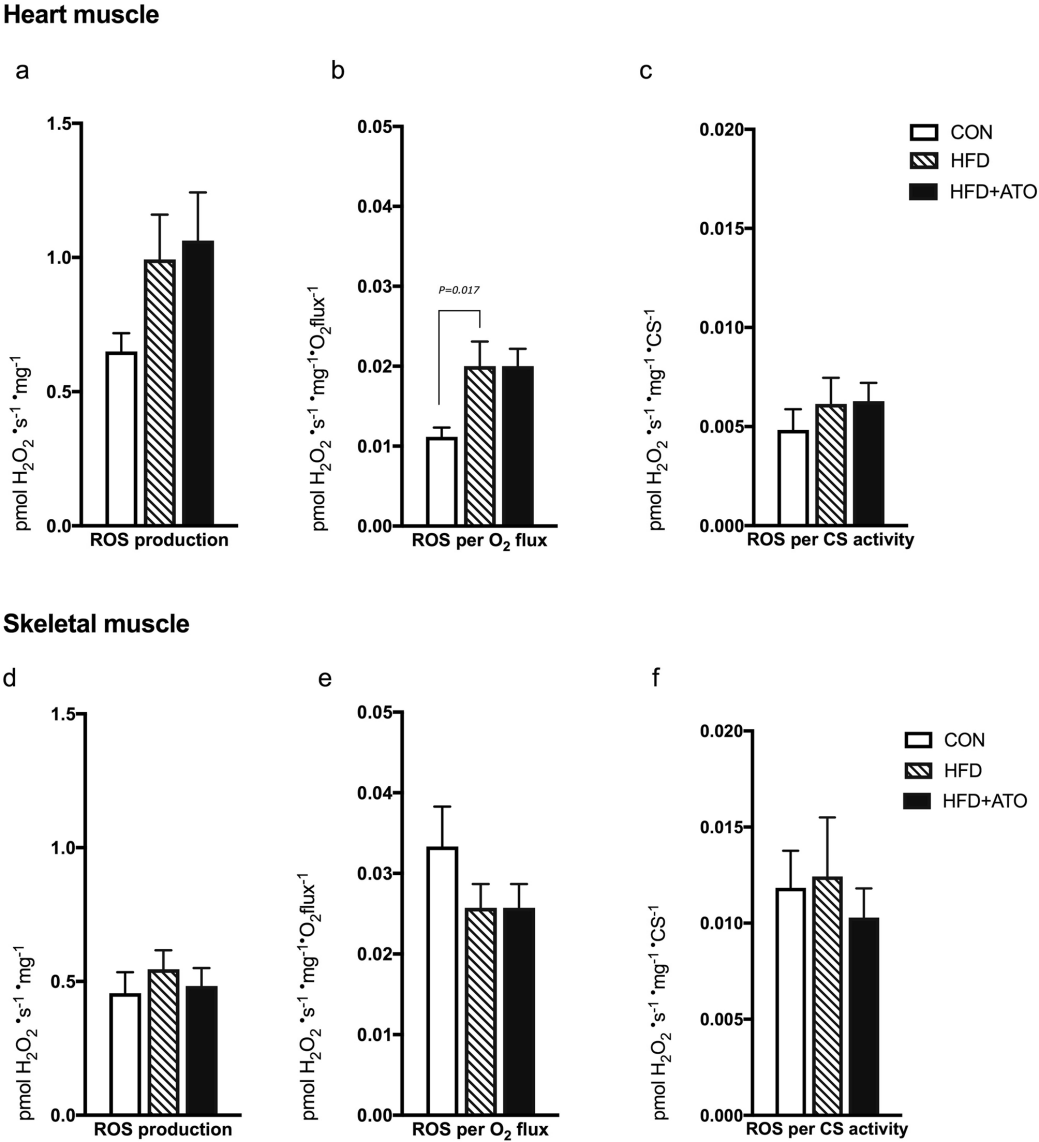
H_2_O_2_ release in permeabilized heart muscle and skeletal muscle of obese and atorvastatin treated Göttingen Minipigs. (**a**). Absolute mass specific complex I + II (malate + glutamate + succinate) supported H_2_O_2_ release (state 2 without addition of ADP) in permeabilized heart muscle fibers from lean control (CON), obese (HFD) and obese atorvastatin treated (HFD + ATO) Göttingen Minipigs. (**b**) H_2_O_2_ release shown in A normalized to oxygen consumption (pmol/s/mg) in permeabilized heart muscle fibers. (**c**). H_2_O_2_ release as shown in A normalized to mitochondrial content expressed as CS activity in permeabilized heart muscle fibers. (**d**) Absolute mass specific complex I + II supported H_2_O_2_ release in permeabilized skeletal muscle fibers from lean control (CON), obese (HFD, n = 7) and obese atorvastatin treated (HFD + ATO) Göttingen Minipigs. (**e**) H_2_O_2_ release as shown in D relative to O_2_ flux in permeabilized skeletal muscle fibers. (**f**) H_2_O_2_ release as shown in D normalized to mitochondrial content expressed as CS activity in permeabilized skeletal muscle fibers. *CS* citrate synthase. Data are means ± SEM, n = 6–7 in each group.

H_2_O_2_ release was measured simultaneously with mitochondrial respiration stimulated by complex I and complex II substrates without adenylates in heart and skeletal muscle. No significant differences between the HFD + ATO and HFD group in H_2_O_2_ release in skeletal and heart muscle were observed (Fig. 6a–f).

The absolute maximal H_2_O_2_ release per mg of wet weight tissue was significantly higher in heart muscle compared to skeletal muscle (*P* < 0.0001, Fig. 6a,d). When expressed relative to mitochondrial respiratory capacity and when normalized to CS activity, H_2_O_2_ release production was significantly higher in skeletal muscle compared to heart muscle (*P* = 0.0007 corrected for mitochondrial respiration, *P* < 0.0001 corrected for CS, Fig. 6b,c,e,f).

Analyses of group differences revealed a significantly increased H_2_O_2_ release in heart muscle in the obese HFD group compared to lean CON minipigs when H_2_O_2_ release was corrected for mitochondrial flux (*P* = 0.017, Fig. 6b).

### Oxidative stress

**Figure 7:**
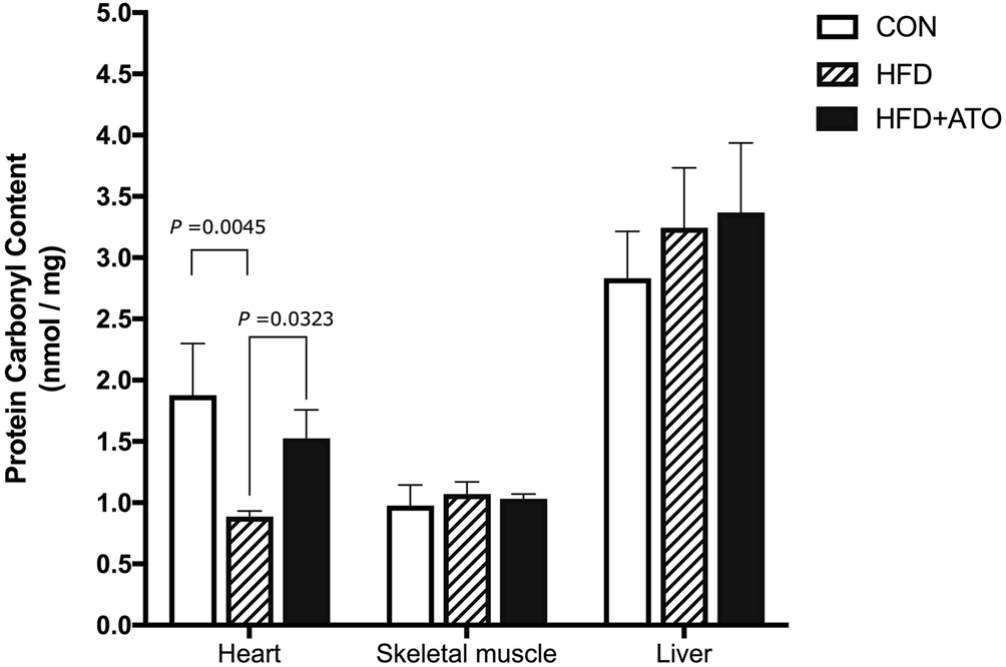
Protein Carbonyl content (PCC) (marker of oxidative stress) measured by fluorometry in heart muscle, skeletal muscle and liver from control (CON), obese (HFD) and obese atorvastatin treated Göttingen Minipigs (HFD + ATO). In heart muscle, PCC was significantly higher in the HFD + ATO group compared to the HFD group (*P* = 0.0323). No significant differences in PCC were found between the groups in skeletal muscle and liver. Bars and error bars denote means ± SEM, n = 6–7 in each group.

Protein carbonylation is a product of elevated levels of ROS. To examine accumulated effects of increased H_2_O_2_ release incurred over time, levels of carbonylated proteins from tissue homogenates of heart, skeletal muscle and liver were determined. In heart muscle, the HFD + ATO had significantly higher protein carbonyl content (PCC) compared to the HFD group (*P* = 0.0323, Fig. 7). Also in the heart, significantly lower PCC was found in the HFD relative to the CON group (*P* = 0.0045). In skeletal muscle and liver, PCC was not found to be significantly different between the groups (Fig. 7).

## Discussion

The main finding of the present study was an impaired mitochondrial respiratory capacity in the liver of obese Göttingen Minipigs when treated chronically with atorvastatin at a clinically relevant oral dose. This finding was accompanied by the detection of atorvastatin and two active metabolites hereof in the liver tissue and in plasma. In the heart and skeletal muscle from the same animals there was no detectable concentration of atorvastatin or its metabolites. There were no statistically significant changes of the mitochondrial function in these tissues but there was a tendency towards opposite directed responses to atorvastatin treatment in the skeletal muscle versus the heart.

The impaired respiratory capacity in liver mitochondria was independent of a cholesterol-lowering effect as the total circulating cholesterol concentration was not affected by atorvastatin treatment in the minipigs. This is in agreement with previous studies using diet-induced obese minipigs and likely relates to the continuous supply of cholesterol in the diet^10,29,30^. In human patients, the recommended dose of atorvastatin for lowering of cholesterol is 10–80 mg per person daily^31^ corresponding to a dose of 0.1–1 mg kg^−1^ body weight. We used approximately 0.5 mg kg^−1^ in the minipigs (35 mg atorvastatin per animal), corresponding to a clinically relevant human dose. When treating with a dose of 10 mg kg^−1^ of atorvastatin, Burnett et al. did show a cholesterol-lowering effect of statins in diet-induced obese minipigs of an undefined breed^32^. The authors did not report on adverse reactions to atorvastatin at this high dose. In our hands, a dose of 35 mg of atorvastatin daily appeared to be the maximum tolerable dose in the Göttingen Minipigs and treatment had to be slowly titrated. Some of the pigs developed adverse reactions (inappetence, staggering gait, increased muscle enzymes) that disappeared when discontinuing treatment.

Mitochondrial dysfunction in the liver has previously been associated with statin therapy in rodent models. In isolated liver mitochondria from diet-induced obese rats treated for 30 days with atorvastatin at 30 mg kg^−1^, the mitochondrial function was impaired^33^. Uličná et al*.* analyzed the mitochondrial respiratory capacity in isolated liver mitochondria from rats following 4 weeks of treatment with atorvastatin at 10 mg kg^−1^ and 80 mg kg^−1^, respectively^34^. They found that the mitochondrial respiratory capacity (supported with complex I linked substrates) was impaired in liver only when using the high atorvastatin dose.

In the present study, treatment with atorvastatin at only 0.5 mg kg^−1^ in minipigs was associated with impaired mitochondrial respiratory capacity with complex I linked substrates and with combined complex I + II substrates a well as uncoupled respiration. The reasons for liver mitochondria from minipigs being more sensitive to statins in the present study, as compared to the previous research in rats, may be several, including different circulatory and metabolic parameters. The amount of drug per kg of BW necessary to reduce cholesterol is higher in rats compared to minipigs^32,34^ and has been suggested to be due to species differences in statin pharmacokinetic properties^35^. Notably, the treatment intervention in rats performed by Uličná et al. was followed for only 4 weeks compared to the 28 weeks intervention in the present study using minipigs. It is possible that the concentration of atorvastatin and its active metabolites in the liver and in plasma of the minipigs resulted from the extended treatment period and that this also led to significant impairment of the mitochondrial respiratory capacity in the liver. The previous literature regarding tissue concentration and circulating concentrations of atorvastatin following in vivo treatment is scarce. In agreement with our results, it has been shown in rats treated with other types of statins that the drug content increases more in the liver than in skeletal muscle^36^. To the best of our knowledge the present study is the first investigating mitochondrial respiratory capacity in liver, skeletal muscle and heart muscle after a longer treatment period using a clinically relevant dose of atorvastatin. The latency period for development of drug induced liver insufficiency after initiation of statin treatment in human patients were reported to be at least 34 days and with a median period of 57, 90 and 155 days, respectively reported in three studies^37–39^. Our results raise the hypothesis that concentration of atorvastatin in the liver, resulting from an extended treatment period, associates with impaired mitochondrial respiratory capacity. This hypothesis deserves a longitudinal study assessing the mitochondrial function at several time points following initiation of statin therapy.

Previous experiments in rats have shown that the mitochondrial respiratory capacity in heart and skeletal muscle cells, responded differently to acute statin treatment in vitro^21^. The authors suggested that tissue specific expression of the monocarboxylate transporter 4 (MCT4) accounts for a higher intracellular concentration of statin in skeletal muscle than heart causing the mitochondria in muscle to be the most sensitive to statin treatment^21^. Our finding of no detectable concentration of statin in heart and skeletal muscle after in vivo treatment does not support this hypothesis in the minipig model. Atorvastatin and active metabolites hereof were available in the circulating blood of treated minipigs, indicating exposure of the drug to the heart and skeletal muscle but uptake into the cells in these organs could not be demonstrated. The minipigs were treated with their last dose of atorvastatin on the day before sampling of plasma and tissue. The interval from the last dose to sampling was not standardized and the atorvastatin concentrations do therefore reflect the presence of atorvastatin but not the maximal concentration. Of notion is that experiments using in vitro treatment of cells with statin reach actual concentrations that correspond to thousand-fold higher concentrations compared to in vivo serum concentrations in human patients^35^. In skeletal muscle, our results showed a tendency for impaired mitochondrial respiratory capacity whereas in heart there was a tendency towards enhanced mitochondrial respiration, following atorvastatin treatment in minipigs. Interestingly, this was in accordance with a previous study, showing that the mitochondrial function in heart muscle mitochondria was enhanced following statin therapy^22^. We cannot conclude that an opposite directed effect of statin on heart and skeletal muscle respiratory capacity occurs, as suggested previously^22^ but our findings in a limited number of animals appear to support this hypothesis.

Our results did not confirm the previous results reported by Bouitbir et al. showing that in skeletal muscle H_2_O_2_ emission increased severely in response to atorvastatin therapy while in the heart there was either a small increase in H_2_O_2_ emission (in vitro model) or a decrease (human patients) after statin treatment^22^. H_2_O_2_ release, oxidative stress expressed as protein carbonylation and oxidation rate of Q10, respectively, as well as the concentration of total Q10 in the skeletal muscle were unaffected in the HFD + ATO group compared to the HFD group. Other studies are in agreement with our results showing no increase in H_2_O_2_ release and no change of the Q10 concentration after statin therapy in skeletal muscle^40,41^.

In the heart muscle, PCC did increase significantly in the HFD + ATO group compared to HFD, suggesting an increase in oxidative stress in the heart muscle with atorvastatin therapy. This finding was not substantiated by any changes in oxidation rate of Q10, total Q10 concentration, H_2_O_2_ release or impaired respiratory capacity. An enhancing and preconditioning effect by atorvastatin on heart muscle mitochondria, as suggested previously in vitro in rat heart muscle cells^22^ was therefore not justified in vivo in the present study.

When showing the opposite directed tissue specific changes in mitochondrial function in response to in vivo atorvastatin treatment, Bouitbir et al. obtained heart and skeletal muscle biopsies that were not obtained from the same patient and the patients were receiving other medication than statin for comorbidities such as heart failure and diabetes. Statin type and doses were also not standardized and this was limiting for the direct comparison of the effects exerted by statin therapy on mitochondrial function in heart versus skeletal muscle^22^. Access to paired heart and skeletal muscle biopsies from human patients is limited making large animal models such as the Göttingen Minipig model of obesity and atherosclerosis very relevant to study tissue-specific responses of mitochondria to various drugs.

The inclusion of a non-obese control group enabled us to observe modifications in the mitochondrial function and oxidative stress related to obesity and compare these with changes induced by atorvastatin therapy. Obesity is linked to tissue specific alterations of mitochondrial function in liver, heart and skeletal muscle^42^. Complex I linked mitochondrial respiration increased significantly in skeletal muscle of the HFD group of minipigs compared to the CON group but when normalizing for mitochondrial content, the difference was not statistically significant. Although not reaching statistical significance, our data indicated an increase in mitochondrial respiratory capacity supported with fatty acid and complex I + II substrates in skeletal muscle of obese minipigs which is in line with previous studies using rodent models of diet induced obesity^43–45^. The literature is, however, ambiguous concerning the direction of changes in mitochondrial function in obese versus lean subjects. Others have demonstrated either no significant change^33^ or decreases^46^ in mitochondrial respiratory capacities in muscle from obese patients compared to lean control subjects. Especially, the role of insulin sensitivity driving mitochondrial dysfunction is debated in the literature (reviewed by Sergi et al.^47^). A previous study showed a decrease in insulin sensitivity in the Göttingen Minipig model of obesity after feeding a high fat diet for 43 weeks compared to lean control^29^. It is likely that the HFD group in the present study had similarly impaired insulin sensitivity although this was not measured at the time of termination. While the mitochondrial respiratory capacity increased numerically in the skeletal muscle from the HFD group compared to CON, there are no indications in this animal model that impaired insulin sensitivity drives mitochondrial dysfunction. Future studies designed to correlate insulin sensitivity and mitochondrial respiration in muscle should be conducted to explore this relationship further.

The obese minipigs had significantly reduced mitochondrial respiratory capacity in the liver compared to their lean counterparts. This indicates that our finding of impaired mitochondrial function in the obese minipigs treated with statin seemingly represents a further aggravation of an already compromised mitochondrial respiratory capacity in the liver in obese animals. The minipigs in the HFD group had severe hepatomegaly as previously described for this model^29,48^. A previous study showed histological changes in this animal model compatible with a NAFLD like phenotype with fibrosis, inflammation and cytoplasmic vacuoles but without significant macro- and micro-vesicular steatosis consistent with NASH^25^.

Previous studies in animal models and humans have investigated the influence of obesity on liver mitochondrial function but the results are controversial^42^. In mice with diet- induced obesity and fatty livers the liver mitochondrial function was impaired^49,50^. Our results, showing a decrease in mitochondrial respiratory capacity supported by complex I and complex II in HFD minipigs compared to CON are in accordance with this finding. Accompanying the reduced respiratory capacity in the liver of obese minipigs was a significant decrease in total Q10 concentration in liver tissue samples and a significant lowering of the proportion of oxidized Q10 compared to the lean CON group. The presence of fatty liver disease could cause a reduction of endogenous Q10, as the liver is the main site for Q10 synthesis^51^. The shift towards a larger proportion of reduced Q10 in the liver of HFD minipigs may be interpreted as a compensatory mechanism towards an increased demand of antioxidant capacity as a response to oxidative stress^52^. Despite the compromised respiratory capacity in liver of HFD + ATO compared to HFD minipigs, no changes in the concentration of Q10 between the two groups were observed in the liver. A significant decrease in oxidation rate was also seen in the heart of the HFD group compared to CON and in skeletal muscle of the HFD + ATO group compared to the HFD group. At this point, however, this oxidative stress mechanism is only speculative and not supported by increased oxidative stress levels in the tissues.

In human patients, two studies investigated mitochondrial respiratory capacity in obese patients versus lean control patients and reported different results^53,54^. Enhancement of the mitochondrial respiratory capacity in obese patients with or without NAFLD relative to lean control patients was reported in one study and it was suggested by the authors to be related to the enhanced substrate supply in obese patients^54^. In the same study, after progression to NASH, the mitochondrial respiratory capacity was significantly impaired despite an increase in CS activity^54^. Lund and colleagues, on the other hand, reported a preserved mitochondrial respiratory capacity and no change in CS activity in the liver of obese patients with NAFL compared to control patients^53^. Although a similar classification system was used in the human studies^55^, the discrepancy in the results of mitochondrial respiration could be due to the patients being in different stages of NAFLD^42^.

Some limitations and advantages apply to this study. If we were to run three high-resolution respirometry protocols in each of the three tissues: liver, heart and skeletal muscle, the maximum capacity of the laboratory would be exceeded. H_2_O_2_ release was therefore measured only in heart and skeletal muscle but not in the liver. The production of ROS in a cell is matched by antioxidant systems. Nevertheless, fluxes in ROS levels are frequent and have signal propagating properties. Alterations of ROS levels are therefore not inherently pathogenic. However, if the levels of ROS are amplified beyond the compensatory thresholds of the antioxidative systems, ROS molecules can mediate damage to biological molecules. Carbonylation of proteins are an example of such damage. In this study H_2_O_2_ was determined as a measure of present ROS levels, while PCC was measured as a measure of accumulated damages inferred by long term ROS fluctuations. Significant changes between the groups of minipigs that were observed for H_2_O_2_ release and PCC, respectively were not unidirectional. This could suggest that the altered H_2_O_2_ release has a role in signal transduction rather as a damaging event. However, this is outside the scope of this work.

The number of animals terminated per day in the larger study that the animals were part of, exceeded the number of animals on which high resolution respirometry could be performed each day. This limited our group sizes and may have caused a lack of power in order to observe significantly opposite directed changes in the mitochondrial respiratory capacity of heart and skeletal muscle in response to atorvastatin therapy. Despite the limited number of animals, we have generated novel insight regarding atorvastatin induced changes of mitochondria in the liver and regarding the detection of atorvastatin in different types of tissues in this species.

An important strength of this study was the use of tissue samples from the heart, skeletal muscle and liver from the same animals. In addition to that, the minipigs were treated with atorvastatin in a standardized dose in vivo as opposed to previous studies investigating the influence of statin therapy in heart and skeletal muscle but using tissues obtained from different patients^22^.

The novel finding of atorvastatin reducing mitochondrial respiratory capacity in the liver of minipigs suggests the need for studies in human patients investigating if similar subcellular mechanisms are observed with chronic statin therapy and how this affects the long term clinical outcome. As a group of lean minipigs treated with atorvastatin was not included in the present study, it needs to be determined if our finding of mitochondrial dysfunction and of atorvastatin and its metabolites being present in the liver were exacerbated by the occurrence of NAFLD. Statins, due to their effectiveness in reducing liver fibrosis, steatosis, and disease progression, are predicted as promising drugs for treatment of patients with NAFLD in the future^16^.

With NAFLD being an increasing health problem worldwide, the use of statins in this disease is likely to increase, although results on the clinical response in large, prospective randomized clinical trials are still pending.

In conclusion, we demonstrate in the present study that atorvastatin and its metabolites can be detected in plasma and the liver but not in heart and skeletal muscle after oral treatment with atorvastatin in obese Göttingen Minipigs. In conjunction with this finding, atorvastatin treatment impairs the respiratory capacity in liver mitochondria. Our results suggest that obesity by itself reduces mitochondrial function in liver and an additional impairment is induced with atorvastatin treatment in this animal model. No direct link was established between impaired mitochondrial function in liver with atorvastatin treatment and oxidative stress and coenzyme Q10 levels.

## Methods

### Animal model

The study was approved by The Animal Experiments Inspectorate, The Danish Ministry of Environment and Food and all procedures conformed with the EU directive on protection of Animals for Research Purposes Directive 2010/63/EU. The study was carried out in compliance with the ARRIVE guidelines.

Twenty castrated male Göttingen Minipigs (Ellegaard Göttingen Minipigs A/S, Dalmose, Denmark) that were a subpopulation of a larger cohort of 80 Göttingen Minipigs, were included at a mean age of 28 weeks (SD ± 1 week).

The animals were housed at the experimental animal unit at the University of Copenhagen in controlled conditions (temperature, 22–24 °C, relative air humidity of 50–70% at a natural day/night cycle. They were group housed, except for periods when animals had central venous catheters implanted. They had free access to bedding material and water.

Lean control animals (CON, n = 6) were fed a standard mini pig chow (Mini-Pig Expanded, Special Diets Services, Witham, UK) according to the breeder’s recommendations.

The high fat diet group (HFD, n = 7) was fed a high-fat high-fructose and high-cholesterol (2%) diet (9G4U) for 5 months and subsequently a similar high cholesterol diet (1%) (5B4L) for the remaining 8 months (both from TestDiet, St. Louis, Missouri, US). The minipigs were fed 2% of body weight but maximum 1000 g/day initially and after 11 months the amount was reduced to 500 g/day due to severe obesity and lameness among the minipigs.

A group of minipigs (HFD + ATO) was fed the high fat high cholesterol diet as described for the HFD group and superimposed with oral treatment with atorvastatin (Atorvastatin, Actavis A/S, Dublin, Ireland) from the 30th study week, provided daily with the feeding, in an apple. The dose of atorvastatin was titrated up to 35 mg/pig/day over 4 weeks and thereafter treatment was continued until termination of the study in week 58 after study start. If animals became anorectic, atorvastatin treatment was discontinued and reintroduced at a lower dosing after starting eating again. A schematic presentation of the study design is shown in supplementary material (Supplementary material Fig. S2).

### Phenotypic characterization

Body weight measurements were performed weekly throughout the study.

One to 2 weeks prior to termination, the animals were full-body scanned using dual X-ray absorptiometry (DXA, GE Lunar Prodigy, GE Healthcare, Brøndby, Denmark) and blood samples were obtained for triglyceride and total cholesterol measurements as previously described^24^ . On the day of termination, blood samples were drawn prior to induction of anesthesia from the cervical vein into one EDTA and one plain tube. The blood was delivered to The Veterinary Diagnostic Laboratory, (University of Copenhagen, Department of Veterinary Clinical Sciences, Frederiksberg, Denmark) for a complete blood count and serum biochemistry analyses.

### Tissue sampling

At termination of the study, the minipigs were fasted overnight. A surgical anesthesia was obtained using tiletamin and zolazepam mixture (Zoletil 50 Vet, ChemVet, Silkeborg, Denmark) with added ketamine (Ketaminol Vet (100 mg/mL) Intervet, Skovlunde, Denmark), xylazine (0.84 mg/kg) (Rompun Vet (20 mg/mL) Bayer, Lyngby, Denmark) and buthorphanol (0.16 mg/kg) (Torbugesic (10 mg/mL) Scanvet, Fredensborg, Denmark).

Then the pigs were euthanized by exsanguination and immediately thereafter the organs were harvested, weighed and sampled for analyses.

For this study a tissue sample of approximately 2 × 1 cm was cut from the left ventricular wall of the heart, transmurally and distal to the coronary artery branches. From the liver, a tissue sample of similar size was obtained from the medial lobe and beneath the capsule. A skeletal muscle sample, also of similar size was obtained from *m. vastus lateralis* on the right rear leg.

From each of the three tissue biopsies, one portion was snap-frozen in liquid nitrogen and stored at − 80° until analysis. Samples of approximately 20 mg from heart, liver and skeletal muscle were placed in separate vials containing ice-cold preservation buffer (BIOPS, see supplementary material for content), and placed on wet ice for transport to the laboratory for immediate analyses of mitochondrial respiratory capacity and H_2_O_2_ release.

### Mitochondrial respiratory capacity and H_2_O_2_ release

The mitochondrial respiratory capacity was measured using high resolution respirometry (Oxygraph O2K, Oroboros, Innsbruck, Austria). In heart and skeletal muscle H_2_O_2_ release was measured simultaneously using the O2k-Fluorometer (OROBOROS O2k-Fluorometer, Oroboros, Innsbruck, Austria) as previously described^56^. Samples from liver were gently dissected with sharp scissors, as previously described^53^. Permeabilized heart and skeletal muscle fibers were prepared as previously described^57,58^. In brief, muscle fibers were manually dissected under a magnifying lens, using 23G needles in ice-cold preservation buffer (BIOPS for biopsies determined to be used for mitochondrial respiration and Buffer X for biopsies determined for measurement of H_2_O_2_ release, see supplementary material for content). Then the fiber bundles were permeabilized by gentle agitation for 30 min in ice-cold BIOPS or buffer X, respectively, with addition of 50 µg/mL of saponin^59^ and rinsed twice by agitation for 10 min in ice-cold respiration medium (MiR05 for mitochondrial respiration measurements, buffer Z for measurement of H_2_O_2_ release. See supplementary material for content). ROS production was measured in state 2 (no ADP added) supported with complex I + II substrates. The H_2_O_2_ release was measured with Amplex red in the presence of horseradish peroxidase as previosuly described^56^. In brief, H_2_O_2_ reacts with Amplex red in a 1:1 relationship to form resorufin, a stable fluorescent compound. Addition of superoxide dismutase (SOD) converts released superoxide into H_2_O_2_. Resorufin production is detected as an increase in fluorescence intensity and the time derivative equals the fluorescence release (ΔF × s^−1^). The protocol is described in supplementary material. Background fluorescence was subtracted and the standard curve established for each measuring condition was used for the calculation of H_2_O_2_ release. The maximal H_2_O_2_ release was expressed relative to mg of wet tissue weight, relative to mitochondrial respiration and relative to CS activity.

Samples weighing between 1.5 and 2 mg for heart, 2–3 mg for skeletal muscle and 2–3 mg for liver were added to each respirometry chamber. All measurements were done in duplicates, with a chamber temperature of 37 °C and hyperoxygenation (450–200 nmol/mL) to avoid oxygen limitations.

A total of eight oxygraphs were run in parallel at a time and three substrate–uncoupler–inhibitor titration protocols were used (see supplementary materials for protocols). Data were retrieved using software (Datlab 6 Oroboros Instruments, Innsbruck, Austria). The intrinsic mitochondrial respiratory capacity was estimated as mitochondrial respiratory capacity normalized to CS activity.

### Enzyme activities

In humans, a correlation between the activity of the citric acid enzyme citrate synthase (CS) and tissue mitochondrial content has been established in skeletal muscle biopsies from healthy, adult men^60^. CS activity was measured using frozen tissue samples as previously described^61^ with optimization for pig liver, heart and skeletal muscle, respectively. In brief, approximately 2–4 mg (heart), 10–12 mg (liver) or 5 mg (skeletal muscle) wet weight of tissue was homogenized in 800 μL (liver) or 1000 μL (heart or skeletal muscle) of buffer containing K_2_HPO_4_ (0.3 M), 0.05% bovine serum albumin (BSA), pH 7.7 for 2 min on a Tissuelyzer (Qiagen, Venlo, Limburg, the Netherlands). 10% triton was added to a final concentration of 0.1% triton and the samples were left on ice for 15 min before they were stored at − 80 °C for later analysis.

Enzymatic activities are expressed as micromoles substrate per minute per gram of wet weight of tissue.

### Atorvastatin content

The atorvastatin calcium salt (ATO), 2-hydroxy atorvastatin dihydrate monosodium salt (2-OH ATO) and 4-hydroxy atorvastatin disodium salt (4-OH ATO) were quantified in the three different tissue types: liver, skeletal muscle and heart muscle and in plasma (EDTA-stabilized) using ultrahigh-performance liquid chromatography-mass spectrometry (UHPLC-MS) system. For every type of biological material, a calibration curve was prepared individually by spiking working solutions into statin-free specimen (obtained from non-treated animals) in a concentration range between 1.36–50 ng/mL (liver), 0.387–50 ng/mL (skeletal muscle) and 0.876–50 ng/mL (heart muscle) and 0.009–10 ng/mL (plasma). The limit of detection for the each analyte was following: 0.013 ng/mL (ATO in liver), 0.449 ng/mL (2-OH ATO in liver), 0.126 ng/mL (4-OH ATO in liver), 0.014 ng/mL (ATO in skeletal muscle), 0.087 ng/mL (2-OH ATO in skeletal muscle), 0.128 ng/mL (4-OH ATO in skeletal muscle), 0.025 ng/mL (ATO in heart muscle), 0.027 ng/mL (2-OH ATO in heart muscle), 0.289 ng/mL (4-OH ATO in heart muscle), 0.002 ng/mL (ATO in plasma), 0.002 ng/mL (2-OH ATO in plasma) and 0.048 ng/mL (4-OH ATO in plasma). Samples and calibration curves were prepared in the same way. In the first step, tissues were homogenized using a bead mill homogenizer. After that, the extraction was performed with cold (− 20 °C) 50% methanol by storing on ice (30 min), vortex-mixing (1 h) and centrifuging (21,000×*g* for 10 min at 20 °C). In the next step, supernatants were evaporated to dryness under vacuum and reconstituted in 5 mM ammonium formate. In plasma samples protein precipitation and statins extraction were conducted with adding cold (− 20 °C) mixture of methanol and acetonitrile (1:1 v/v). Then samples were vortex-mixed (3 min), incubated on ice (5 min) and centrifuged (21,000×*g* for 10 min at 4 °C). The samples were analyzed by LC–MS system. Chromatographic separation was obtained on Zorbax Eclipse Plus C18 RRHD (2.1 × 50 mm, 1.8 µm, Agilent Technologies) chromatographic column equipped with pre-column (UHPLC Guard 3PK Zorbax Eclipse Plus C18, 2.1 × 5 mm, 1.8 µm, Agilent Technologies). Deionized water with 0.1% formic acid (phase A) and acetonitrile with 0.1% formic acid (phase B) were used as mobile phases. The gradient was changing from 10% phase B at the beginning to 95% phase B at the end of the run. The total run time was 4 min. The Triple Quad (6495 Agilent Technologies) was used as a detector. The mass spectrometer was working on the dynamic multiple reaction monitoring (dMRM) mode. For ATO, 2-OH ATO and 4-OH ATO the following MRM transitions were monitored respectively: 559.1–466.0 and 559.1–440.0, 575.1–466.0 and 575.1–440.0, 575.1–466.0, and 575.1–440.0.

### Protein carbonyl content

The tissue content of protein carbonyls were measured using the OxiSelect Protein Carbonyl Fluorometeric Assay (Cell Biolabs) according to manufacturer instructions. 200 µg of protein lysate from treated skeletal muscle, heart and liver was derivatized with protein carbonyl fluorophore. Proteins were TCA precipitated and free fluorophore was removed by washing with acetone. Pellet was dissolved in guanidine hydrochloride and the absorbance of the protein-fluorophore was measured using a 485485/538-nm filter set in a Fluoroscan Ascent plate reader (Thermo Scientific) and normalized to nmol/mg using a standard curve of known protein carbonyl fluorophore concentrations.

### Coenzyme Q10 concentration

Concentrations of reduced and oxidized Q10 were analysed in tissue samples from heart muscle, skeletal muscle and liver using high-performance liquid chromatography (HPLC) with electrochemical detection (ECD) as previously described with modification^62^. In brief, approximately 30 mg of tissue was mechanically homogenized in 2-propanol and, following centrifugation at 20,900*g* for 2 min at 4 °C, 40 µL of supernatant was injected into a high-performance liquid chromatography (HPLC) apparatus with an electrochemical detector (ECD). In order to reduce the time of analysis, the mobile phase 2 was modified in 50 mM sodium perchlorate in methanol/isopropanol (90/10 v/v) with a flow rate of 0.24 mL/min. Total Q10 was calculated as the sum of reduced and oxidized concentrations of Q10 and expressed as mg/kg tissue. The oxidation rate of Q10 was calculated as the proportion (%) of oxidized Q10 relative to the total concentration of Q10.

### Statistical analyses

Statistical analyses and figures were performed using SAS version 9.4 (SAS Institute Inc., Cary, NC, USA) and Graph Pad Prism Version 8 (GraphPad Software Inc., La Jolla, CA, USA). A *P* value of 0.05 was considered statistically significant.

Group differences between continuous variables reported in Table 1 were analyzed using a one-way ANOVA. Logarithmic transformation was applied to obtain homogeneity of residuals where appropriate. For variables where homogeneity of variation could not be obtained, overall group differences were tested using Kruskall Wallis test and post-hoc paired comparison were done using the Wilcoxon rank-sum test.

A linear model taking repeated measures into account was used to test for differences between the HFD + ATO and HFD groups and between the HFD and CON group, respectively, in parameters of cardiac, skeletal muscle and liver mitochondrial respiratory capacity, H_2_O_2_ release, CS activity, PCC, total Q10 concentration and Q10 oxidations rate. To meet the model assumptions, including homogeneity of residuals on visual inspection, some parameters were log transformed prior to the analyses.

Tissue type and group (CON, HFD and HFD + ATO) were included as class variables and interaction between group and tissue type was evaluated in the statistical model. Animal ID was as random effect parameter.

## Supplementary Information

Supplementary Informations.
