# Supplementary material for: Atorvastatin impairs liver mitochondrial function in obese Göttingen Minipigs but heart and skeletal muscle are not affected
Source: Sci Rep. 2021 Jan 26;11:2167. doi: 10.1038/s41598-021-81846-9 (PMC7838180; doi:10.1038/s41598-021-81846-9)
Supplement: Supplementary file 1 — Supplementary Informations. [file 41598_2021_81846_MOESM1_ESM.pdf]

Supplementary file to:

Atorvastatin impairs liver mitochondrial function in obese Göttingen Minipigs but heart and skeletal muscle are not affected

Journal: Scientific Reports

Liselotte Bruun Christiansen[1*], Tine Lovsø Dohlmann[2], Trine Pagh Ludvigsen[3], Ewa Parfieniuk[4], Michal Ciborowski[4], Lukasz Szczerbinski[4], Adam Kretowski[4], Claus Desler[5], Luca Tiano[6], Patrick Orlando[6], Torben Martinussen[7], Lisbeth Høier Olsen[1], Steen Larsen[2,4*].

[1]The LIFEPHARM Centre, Department of Veterinary and Animal Sciences, Faculty of Health and Medical Sciences, University of Copenhagen, Ridebanevej 9, 1870 Frederiksberg, Denmark
[2]Xlab, Center for Healthy Aging, Department of Biomedical Sciences, Faculty of Health and Medical Sciences, University of Copenhagen, Blegdamsvej 3B, 2200 Copenhagen, Denmark
[3]Global Drug Development, Novo Nordisk A/S, Novo Nordisk Park, 2760 Måløv, Denmark
[4]Clinical Research Centre, Medical University of Bialystok, 15-089 Bialystok, Poland
[5]Center for Healthy Aging, Department of Cellular and Molecular Medicine, University of Copenhagen, Blegdamsvej 3B, 2200 Copenhagen, Denmark
[6]Polytechnic University of Marche, Department of Life and Environmental Sciences (DISVA), via Brecce Bianche, Ancona, Italy
[7]Department of Public Health, Faculty of Health and Medical Sciences, University of Copenhagen, Øster Farimagsgade 5, 1014 Copenhagen, Denmark

*Corresponding authors
Liselotte Bruun Christiansen. Email: lbc@sund.ku.dk

Steen Larsen. Email: stelar@sund.ku.dk

Supplementary figures

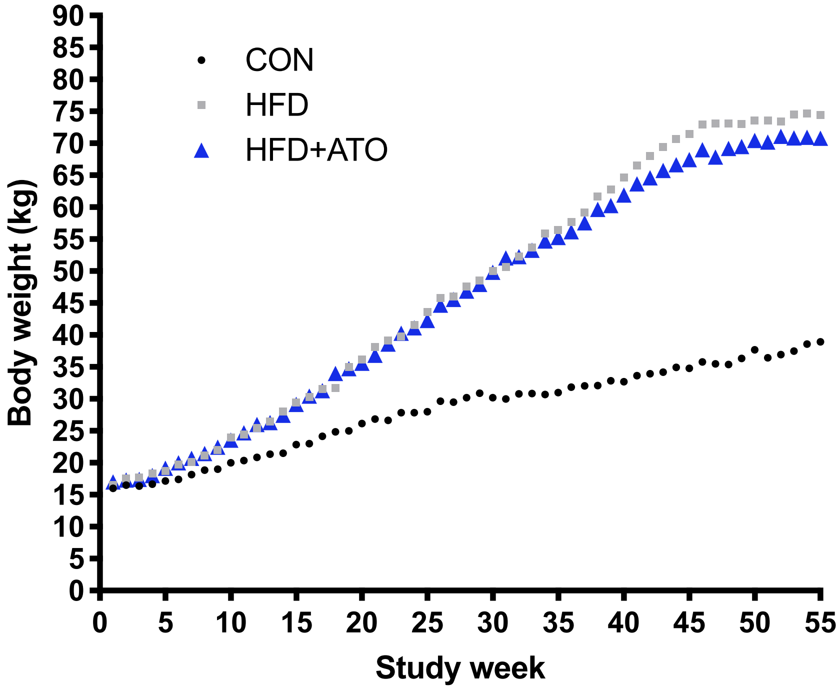

Fig. S1. Increase in body weight (BW) of Göttingen Minipigs during the study period.

At study start, minipigs were of equal BW. Minipigs fed a high fat and high cholesterol diet (HFD, n=7) and minipigs fed a high fat diet and treated with atorvastatin (HFD+ATO, n=7) increased their BW considerably compared to minipigs fed a standard diet (CON, n=6). At termination of the study, HFD and HFD+ATO had significantly higher BW than CON (P=0.0034). Data are means, n=6-7 in each group. The figure is modified from[1].

<table>
<tr><th>Group \ Study month</th><th>1</th><th>2</th><th>3</th><th>4</th><th>5</th><th>6</th><th>7</th><th>8</th><th>9</th><th>10</th><th>11</th><th>12</th><th>13</th></tr>
<tr><td>CON</td><td colspan="13">Standard diet
2% of mean BW until BW 35 kg, then 500g/day</td></tr>
<tr><td>HFD</td><td colspan="5">High fat, high fructose, high cholesterol (2%) diet (5B4L), fed 2% of mean BW</td><td colspan="6">High fat, high fructose, high cholesterol (1%) diet (9G4U), fed 2% of mean BW but max 1000g/day</td><td colspan="2">High fat, high fructose, high cholesterol (1%) diet (9G4U), fed 500g/day</td></tr>
<tr><td rowspan="2">HFD+ATO</td><td colspan="5">High fat, high fructose, high cholesterol (2%) diet (5B4L), fed 2% of mean BW</td><td colspan="6">High fat, high fructose, high cholesterol (1%) diet (9G4U), fed 2% of mean BW but max 1000g/day</td><td colspan="2">High fat, high fructose, high cholesterol (1%) diet (9G4U), fed 500g/day</td></tr>
<tr><td colspan="7"></td><td colspan="6">Atorvastatin titration from 0 to 35 mg/minipig
10 mg increase every week</td></tr>
</table>

Fig. S2. Schematic overview of the study design showing dietary and treatment interventions according to the time of study in Göttingen Minipigs.
CON: Control group, BW: Body weight, HFD: high fat diet group, HFD+ATO: High fat diet and atorvastatin treatment group. 9G4U and 5B4L indicate the manufacturers name of the diets used in the study (TestDiets, Missouri, USA). Figure is modified from[2].

Supplementary methods

The mitochondrial respiratory capacity was measured using high resolution respirometry (HRR, Oxygraph O2K, Oroboros, Innsbruck, Austria).

Buffers for tissue preparation

Preservation buffer "BIOPS" used for permeabilization of skeletal muscle, heart muscle and liver samples, prior to mitochondrial respirometry measurements: final concentrations: (2.8 mM) Ca_2K_2EGTA, (7.23 mM) K_2EGTA, (5.77 mM) Na_2ATP, (6.56 mM) $MgCl_2 \cdot 6H_2O$, (20 mM) taurine, (15mM) Na_2Phosphocreatine, (20 mM) Imidazole, (0.5 mM) dithiotretiol (DTT), (50 mM) potassium salt morpholineethanesulfonate (K^+ MES), pH 7.1)[3].

Buffer X used for permabilization of heart and skeletal muscle samples prior to measurements of H_2O_2 release analyses : 60 mM (K^+MES), 35 mM KCl, 7.23 mM K_2EGTA, 2.77 mM CaK_2EGTA, 20 mM imidazole, 0.5 mM dithiothreitol (DTT), 20 mM taurine, 5.7 mM Na_2ATP, 15 mM Na_2 phosphocreatine, 6.56 mM $MgCl_2 \cdot 6H_2O$, and H_2O).

Substrate inhibitor titration protocols

The mitochondrial respiratory capacity was compared between the three groups (CON, HFD and HFD+ATO) in liver samples and in permeabilized muscle fibers from heart and skeletal muscle. Titration protocols were used to stimulate state 3 respiration (respiration with adenylates) with substrates supporting electron transport through complex I, complex II and convergent electron flow through complex I+II. Moreover, uncoupled respiration and complex IV stimulated respiration were included in the protocols. In heart and skeletal muscle, state 3 respiration supported with the fatty acid substrate palmitoyl carnitine delivering electrons through the electron transfer flavoprotein (ETF) was also determined.

High resolution respirometry and high resolution fluorometry were done with the following respiration buffers:

Respirometry

Mir05: 110 mM sucrose, 60 mM K-lactobionate, 0.5mM EGTA, 0.1% (w/v) bovine serum albumin (BSA), 3 mM MgCl2, 20 mM taurine, 10 mM KH_2PO4, and 20 HEPES; pH 7.1.

Fluorometry:

Buffer Z: 1mM EGTA, 5 mM $MgCl_2 \cdot 6H_2O$, 105 mM K^+MES, 30 mM KCl, 10mM KH_2PO4, 5mg/ml BSA: pH 7.1[4]

Three substrate and inhibitor protocols (Protocols A, B and C) were used in the study. No cytochrome *c* effect was seen in any of the protocols.

Protocol A. Evaluating complex I and complex I+II linked respiratory capacity in liver and H_2O_2 release in heart and skeletal muscle (Fig S3a)

Protocol A was applied to liver and measured in respiration medium Mir05.
In heart and skeletal muscle, H_2O_2 release was measured simultaneously with HRR in buffer Z with the addition of 25 μM blebbistatin[5] (Fig S3b).
In the permeabilized fibers from these tissues the following was added: Amplex Red (5 mmol/L, Molecular probes), Superoxide Dismutase (45 U/mL, Sigma), Horseradish Peroxidase (6 U/mL, Sigma) for establishment of baseline fluorescence signal. The following sequential titrations were in heart and skeletal muscle flanked by a titration of 100mM freshly made H_2O_2:

Malate (2 mM) and glutamate (10 mM) leading to state 2 respiration followed by three titrations of succinate (1 mM) (nonsaturated CII leak), succinate (additional 2 mM (3mM in total)) (nonsaturated CII leak), succinate (additional 14 mmol/L (20 mM in total)) (saturated CII leak). This was followed by ADP (5 mM) for obtaining state 3 respiration supported by complex I + complex II substrates. Finally, uncoupled respiratory capacity was measured with carbonyl cyanide-4-(trifluoromethoxy) phenylhydrazone (FCCP) in steps of 0.25 μM until maximal uncoupled respiration (ETS).

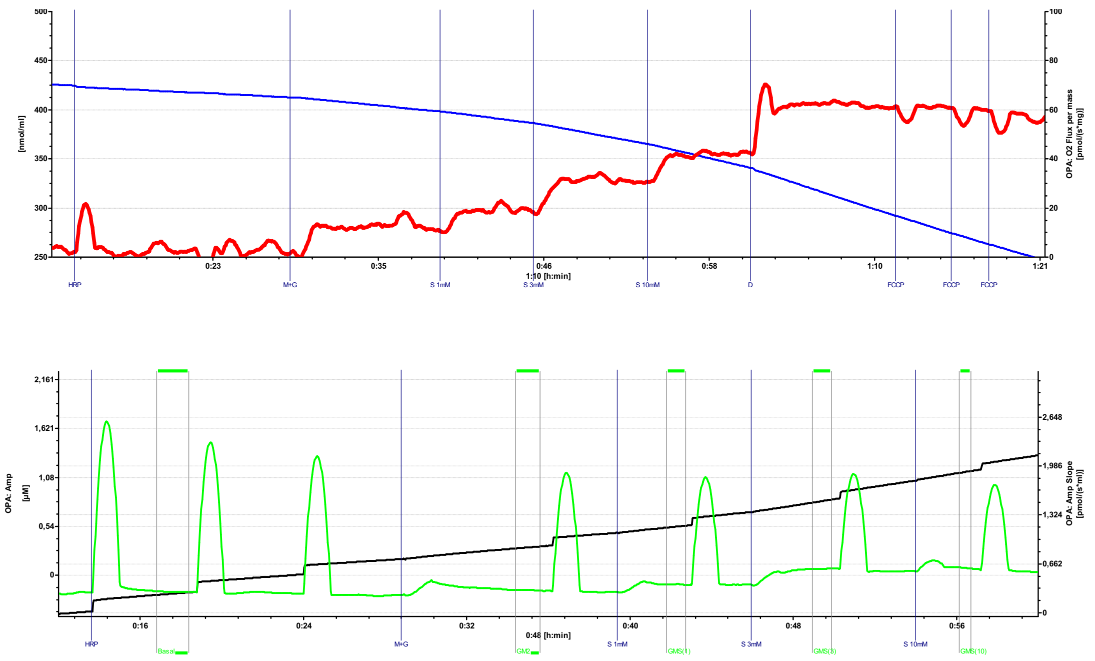

Fig. S3. Representative traces of respirometry (a) and H_2O_2 release (b) using protocol A in heart muscle from Göttingen Minipig in the CON group.

Protocol B: <u>evaluating complex I; complex I+II linked respiratory capacity and COX activity</u>

Protocol B was applied to permeabilized skeletal muscle, heart muscle and to liver using Mir05 as respiration medium:

State 2 respiration (absence of adenylates) was assessed with malate (2 mM) and glutamate (10 mM) followed by state 3 respiration (5 mM ADP) and then cytochrome *c* (10 µM). Simultaneous electron input into complex I + II (maximal OXPHOS capacity) was assessed with succinate (10 mM). Then rotenone (0.5 µM) was added to inhibit complex I, and atractyloside (0,05 mM) to inhibit mitochondrial ADP transport. Then antimycin A (2.5 µM) was added to inhibit complex III (ROX). Finally ascorbate and *N,N,N´,N´*-Tetramethyl-p-phenylenediamine (TMPD) was added (2 mM and 0.5 mM, respectively) to activate complex IV (COX). Figure S4 shows a representative trace of oxygraphic measurements using protocol B in heart

muscle. A chemical calibration was made to correct respiration for auto oxidation of TMPD and ascorbate.

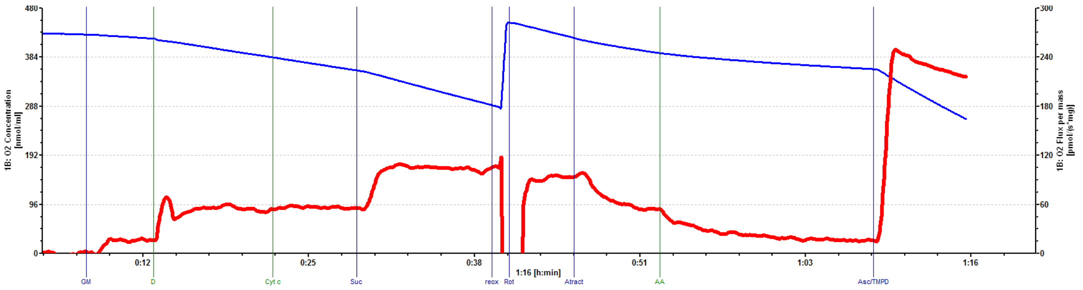

Fig. S4. Representative trace of protocol B in heart muscle from a Göttingen Minipig in the CON group.

Protocol C: <u>Evaluating fatty acid linked respiratory capacity</u>

Protocol C was applied to permeablized skeletal and heart muscle fibers using Mir05 as respiration medium and with the addition of 25 µM blebbistatin.

State 2 respiration was assessed by addition of malate (2 mM) and glutamate (10 mM), nonsaturated state 3 respiration was achieved by adding ADP (0,5 mM). This was followed by the addition of palmitoyl carnitine (0.075 mM) and thereafter ADP (4.5 mM) to obtain maximal coupled respiration with convergent electron input to complex I and electron transfer flavoprotein (ETF) Integrity of the outer mitochondrial membrane was tested by adding cytochrome *c* (10 μM). Representative trace of protocol C applied in heart muscle is shown in Figure S5.

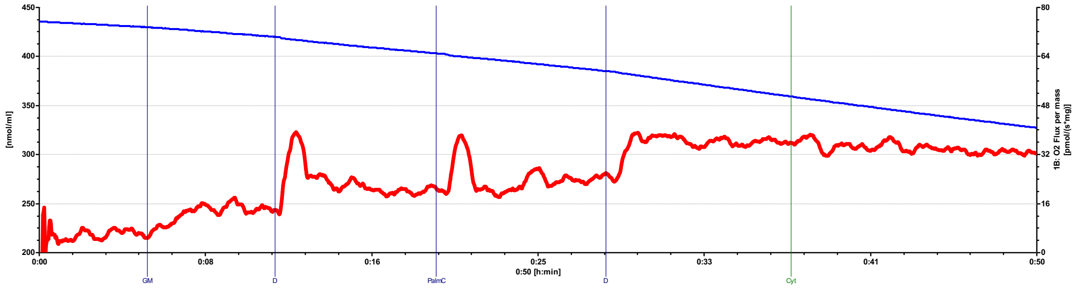

Fig. S5. Representative trace of protocol C applied to heart muscle in a Göttingen Minipig in the CON group.

References

[1] Andreasen, L. J. *et al.* Dietary normalization from a fat, fructose and cholesterol-rich diet to chow limits the amount of myocardial collagen in a Göttingen Minipig model of obesity. *Nutr. Metab.* **15**, 64 (2018). https://doi.org/10.1186/s12986-018-0303-x.

2 Schumacher-Petersen, C. *et al.* Experimental non-alcoholic steatohepatitis in Göttingen Minipigs: consequences of high fat-fructose-cholesterol diet and diabetes. *J. Transl. M*ed. **17**, 110 (2019). https://doi.org/10.1186/s12967-019-1854-y.

[3] Veksler, V. I., Kuznetsov, A. V., Sharov, V. G., Kapelko, V. I. & Saks, V. A. Mitochondrial respiratory parameters in cardiac tissue: A novel method of assessment by using saponin-skinned fibers. *Biochim. Biophys. Acta-Bioenerg.* **892**, 191–196 (1987).

[4] Perry, C. G. R., Kane, D. A., Lanza, I. R. & Neufer, P. D. Methods for assessing mitochondrial function in diabetes. *Diabetes* **62**, 1041–1053 (2013).

[5] Perry, C. G. R. *et al.* Inhibiting myosin-ATPase reveals a dynamic range of mitochondrial respiratory control in skeletal muscle. *Biochem. J.* **437**, 215–222 (2011).
